# A Dataset for Visual Navigation with Neuromorphic Methods

**DOI:** 10.3389/fnins.2016.00049

**Published:** 2016-02-23

**Authors:** Francisco Barranco, Cornelia Fermuller, Yiannis Aloimonos, Tobi Delbruck

**Affiliations:** ^1^Research Center on Information and Communication Technologies (CITIC), Department of Computer Architecture and Computer Technology, University of GranadaGranada, Spain; ^2^Computer Vision Lab, University of Maryland Institute for Advanced Computer Studies (UMIACS), Department of Computer Science, University of MarylandCollege Park, MD, USA; ^3^Department of Information Technology and Electrical Engineering, Institute of Neuroinformatics, ETH Zurich and University of ZurichZurich, Switzerland

**Keywords:** event-driven methods, frame-free sensors, visual navigation, dataset, calibration

## Abstract

Standardized benchmarks in Computer Vision have greatly contributed to the advance of approaches to many problems in the field. If we want to enhance the visibility of event-driven vision and increase its impact, we will need benchmarks that allow comparison among different neuromorphic methods as well as comparison to Computer Vision conventional approaches. We present datasets to evaluate the accuracy of frame-free and frame-based approaches for tasks of visual navigation. Similar to conventional Computer Vision datasets, we provide synthetic and real scenes, with the synthetic data created with graphics packages, and the real data recorded using a mobile robotic platform carrying a dynamic and active pixel vision sensor (DAVIS) and an RGB+Depth sensor. For both datasets the cameras move with a rigid motion in a static scene, and the data includes the images, events, optic flow, 3D camera motion, and the depth of the scene, along with calibration procedures. Finally, we also provide simulated event data generated synthetically from well-known frame-based optical flow datasets.

## 1. Introduction

Asynchronous frame-free vision sensors have gained popularity among vision researchers in recent years. The most prominent of these sensors are the temporal change threshold imager (Mallik et al., 2005), the DVS (Lichtsteiner et al., 2008), the ATIS (Posch et al., 2011), and the DAVIS (Brandli et al., 2014). Inspiration for their design comes from the transient pathway of primate vision, which processes information due to luminance changes in the scene (Lichtsteiner et al., 2008; Liu et al., 2015). Their properties, such as the high temporal resolution (triggering temporal contrast events with a resolution of a few microseconds), low-bandwidth, low-computational resource requirements, low-latency, and real-time performance, make them interesting for many applications of motion perception. While conventional cameras record image luminance at fixed time intervals, frame-free vision sensors record asynchronously the time and location, where changes in the luminance occur.

Visual motion analysis for navigation is about relating the observed intensity changes on the imaging device to the 3D scene geometry and the 3D motion of the observer (or imaging device) relative to the scene. The computational analysis involves two distinct processes: the estimation of observed image motion on the imaging surface due to the movement of scene points, in Computer Vision usually called *optical flow*, and the estimation of the geometry and dynamics of the scene on the basis of image motion. Visual navigation, in general, involves moving cameras in environments that can be dynamic as well, and it refers to a set of tasks ranging from obstacle avoidance, over object tracking, 3D motion estimation and scene segmentation, to map making. Currently, however, our dataset has static scenes only. We provide the raw data along with the 3D motion and the scene geometry, and this data allows for evaluating algorithms concerned with the classic *structure from motion* problems of image motion estimation, 3D motion estimation, reconstruction, and segmentation by depth.

Evaluation datasets drive applications and challenge researchers to develop techniques that are widely applicable, consider diverse scenarios, and have high accuracy. The Computer Vision community has realized their importance for many years, and has provided datasets for many applications, including visual navigation. Among the best known datasets for image motion one can find Middlebury (Baker et al., 2011), MPI Sintel (Butler et al., 2012), and KITTI (Geiger et al., 2012). Middlebury, a benchmark that also provides a creative ranking of methods, has been the standard until the last few years. The more recent MPI Sintel and KITTI datasets include scenarios of greater complexity and much larger image motion. The former consists of synthetic sequences and has many challenging cases such as transparencies, blurring, or variations in illumination. The latter has sequences from real-world driving scenarios, and provides besides optical flow also ground-truth for 3D motion, structure, and the tracking of objects. Other well-known data sets for 3D motion and structure include the CMU dataset (Badino et al., 2015), the TUM dataset (Sturm et al., 2012), as well as the KITTI dataset (Geiger et al., 2012). These datasets were designed for evaluation of navigation and localization algorithms.

Along with datasets, we also need metrics to evaluate the techniques. The metrics of Computer Vision focused mostly on accuracy. Image motion is usually evaluated by the average error of either the flow vectors (Otte and Nagel, 1994), or their directions (Fleet and Jepson, 1990). 3D camera motion is evaluated by the average error in the direction of the rotation axis, the angular velocity, and the direction of translation (see Raudies and Neumann, 2012). Clearly, the average error does not capture fully the quality of a method, given the heterogeneity of sequences in the different datasets. In Sun et al. (2014), statistical significance tests provide a way to cope with this problem.

A few of the methods published in the event-based literature included evaluations. Several methods evaluated the accuracy of image motion estimation methods, e.g., (Barranco et al., 2014; Benosman et al., 2014; Censi and Scaramuzza, 2014; Orchard and Etienne-Cummings, 2014; Tschechne et al., 2014) evaluated odometry estimation. However, all these methods used their own datasets. Therefore, so far there is a lack of comparisons between different event-based methods and comparisons to Computer Vision methods. Another paper, which is part of this special issue (Ruckauer and Delbruck, in review) provides a dataset for the evaluation of event-based flow methods and also releases codes for the evaluated methods. However, this work is the first to present a dataset that facilitates comparison of event-based and frame-based methods for 2D and 3D visual navigation tasks.

Our real-time dataset was collected with a mobile platform carrying a DAVIS sensor (Brandli et al., 2014) and an RGB-D sensor (RGB + Depth sensor). The DAVIS sensor provides asynchronous streams of events called DVS events, and synchronous sequences of image frames called APS frames. From the RGB-D sensor we obtain the depth maps of the scene and from the odometry of the platform we obtain the 3D motion. Using the 3D motion and depth, we compute the image motion. In addition to the data, we also provide the code for the calibration of the DAVIS sensor with respect to the RGB-D sensor (using the synchronous frames of the DAVIS), and the calibration between the robotic platform and the DAVIS sensor. We use the same metrics as in conventional methods to evaluate the accuracy of event-driven methods. To account for the sparseness of the event data, we also include a measure of the data density.

The paper is structured as follows: Section 2 describes current datasets of visual navigation from Computer Vision. Next, Section 3 describes how we created the event-based dataset. Section 4 reviews different metrics for evaluation and Section 5 presents some of the sequences of our dataset. Finally, Section 6 concludes the work.

## 2. Datasets in computer vision

Benchmarks, datasets and quantifiable metrics to estimate accuracy are very common in the Computer Vision literature. They have greatly influenced the development of Computer Vision techniques for different applications, and contributed to market solutions in demanding fields such as medical image analysis, autonomous driving, and robotics.

There are a number of benchmarks for visual navigation. Barron et al. (1994) were the first to propose a benchmark and quantitative evaluation of optical flow methods. This dataset of synthetic scenes was then replaced by the Middlebury database (Baker et al., 2011), which contains much more challenging datasets of synthetic and real scenes with objects at different depth causing motion discontinuities. The success of Middlebury may be partly due to its evaluation platform: through a web interface one can upload the results of a motion estimation method for comparison with the state-of-the-art methods. Half of the example sequences are provided with the ground-truth as training set to allow users to tune their methods. For evaluation, authors are instructed to estimate the motion for the remainder of the sequences (the test set) whose ground-truths are not provided, and to submit them through the web application. Then, the methods are ranked according to different error metrics: endpoint error, angular error, interpolation error, and normalized interpolation error. The most recent prominent datasets, MPI Sintel (Butler et al., 2012) and KITTI (Geiger et al., 2012) are much more challenging. They provide long video sequences at high spatial resolution, and the image motion between frames spans a large range of values (even exceeding 100 pixels). Actually, such large displacements between video frames are not amenable to a continuous modeling of the intensity function, but require discrete approaches similar as used for stereo correspondence. The sequences include deformable objects and introduce very complex problems such as transparencies, shadows, smoke, and lighting variations. Masks for motion boundaries and for unmatched pixels are included, and new metrics are described to measure the image motion accuracy in these areas. MPI Sintel, which is generated with a computer graphic model, provides different variations of its sequence, such as with and without motion blur.

Several other datasets provide benchmarks for 3D position and pose estimation. Usually they include sequences of image frames and the corresponding six parameters of the camera motion defined by the rotation and the translation. Some of these datasets also provide corresponding sequences of depth maps and image motion fields. (Raudies and Neumann, 2009) used the earlier created *Yosemite* sequence, a synthetic fly-through sequence over the so-named valley, and created the synthetic *Fountain* sequence with a curvilinear motion for a patio sequence. KITTI (Geiger et al., 2012) provides a dataset for 3D visual navigation, specifically created for autonomous driving. It includes data from a stereo camera rig, a laser scanner, and GPS/IMU signals. The CMU dataset, available at (Badino et al., 2015), uses the same sensors also mounted on a car. The data of the TUM dataset (Sturm et al., 2012) includes images and depth frames captured with an RGB-D sensor (Microsoft Kinect). The ground-truth odometry was estimated from the external camera-based tracking system and the RGB-D sensor data.

## 3. Dataset design

Event-based sensors and frame-based cameras record very different kinds of data streams, and thus to create a benchmark for their comparison is quite challenging. While conventional frame-based sensors record scene luminance, which is static scene information, event-based sensors record changes in the luminance, which is dynamic scene information. Conventional cameras have a higher spatial resolution than event-based sensors, but their temporal resolution is fixed, usually up to ~60 fps (frames per second). In contrast, for frame-free sensors there is no fixed sampling period, which can be as small as a few microseconds. To compare static images to events, a few works (such as Pérez-Carrasco et al., 2013) shook the sensor. This technique, however, is not applicable for visual navigation, as it would introduce too much additional noise. Indeed, we require a conventional sensor and a frame-free sensor collecting data of the same scene. For our dataset we used the DAVIS sensor, which collects both asynchronous brightness-change events and synchronous frames.

The synthetic data in our benchmark was created from existing Computer Vision datasets (Section 3.1), and includes two sets. First, we generated events (Barranco et al., 2014) for the optic flow sequences provided in Baker et al. (2011) and Barron et al. (1994). The such created dataset allows comparison to the large number of existing optic flow techniques in the Computer Vision literature, but it is not accurate due to the lack of ground-truth information (in the original optical flow sequences) in areas occluded between consecutive frames and ambiguities in the depth discontinuities. This problem was overcome in a second dataset which was built from a graphics-generated 3D scene model (Barranco et al., 2015). The real data in our benchmark was collected with a mobile robot carrying a rig on which we mounted a DAVIS sensor and an RGB-D sensor (RGB images plus Depth; Section 3.2). By calibrating the DAVIS sensor with the depth sensor, we obtained the data required for reconstructing the 3D scene model. The simple odometry system, consisting of a gyroscope and an accelerometer, provided the 3D motion ground-truth.

Note, that we computed the motion of the sensor using the odometry of our platform. An alternative, much easier approach to obtain 3D sensor estimates, would be to use an external motion capture system (Voigt et al., 2011). However, motion capture systems are expensive and cannot be used for outdoor scenarios.

Our dataset is available at http://atcproyectos.ugr.es/realtimeasoc/protected/evbench.html. It includes the DAVIS sequences (DVS events and APS frames), the Kinect data (RGB images and depth maps), the generated motion flow fields, and the 3D camera motion (translation and rotation). The code for the different calibration procedures, registrations, and for computing the evaluation metrics, described in the next sections, are available at the software repository https://github.com/fbarranco/eventVision-evbench.

### 3.1. Simulated events from current computer vision datasets

The first dataset was created from the sequences in Middlebury (Baker et al., 2011) by simulating the events on the basis of the ground truth optic flow (Barranco et al., 2014, 2015). Real frame-free sensors trigger an event when the intensity difference at a point exceeds a predetermined value (more exactly when the change in log intensity exceeds a threshold). To simulate this, we first interpolate image frames in time using the optic flow information. Assuming a frame rate of 20 fps the optic flow sequences, we interpolate 50,000 samples between pairs of consecutive frames to achieve a simulated temporal resolution of 1 μs in the DVS. Then events (with exact timestamp) are created, by checking at every position for changes greater than the threshold. However, this simulation only works at image regions due to smooth surfaces, but not at occlusion regions, where usually ground-truth flow is not provided. To perform reconstruction, a 3D model of the scene is required. In its absence we generated our data using the following approximation: we differentiate between occluded regions, which are pixels visible in the previous frame but not the current, and dis-occluded regions, which are pixels not visible in the previous frame, but uncovered in the current frame. Intensity values of occluded regions are obtained from the previous frame and those of dis-occlusions from the subsequent frame. For non-static regions, we assume the same motion for the background and the region. More complex scenarios, including non-regular motion patterns or occluded objects with different motions, are discarded.

The second dataset was created in a way similar to the MPI Sintel dataset (Butler et al., 2012). Using a 3D graphics model of the scene and information on the 3D motion and 3D pose of the camera, we reconstructed the motion flow field and stream of events (Barranco et al., 2015). Specifically, we used the 3D model, the textures, and the 3D motion ground-truth provided by Mac Aodha et al. (2013), which were created using the 3D software and modeling tool Maya (see http://www.autodesk.com/products/maya). We note that for a more realistic simulation, one could additionally add simulated noise on the events using appropriate probability distributions.

### 3.2. DAVIS mounted on a mobile platform

**Figure 1:**
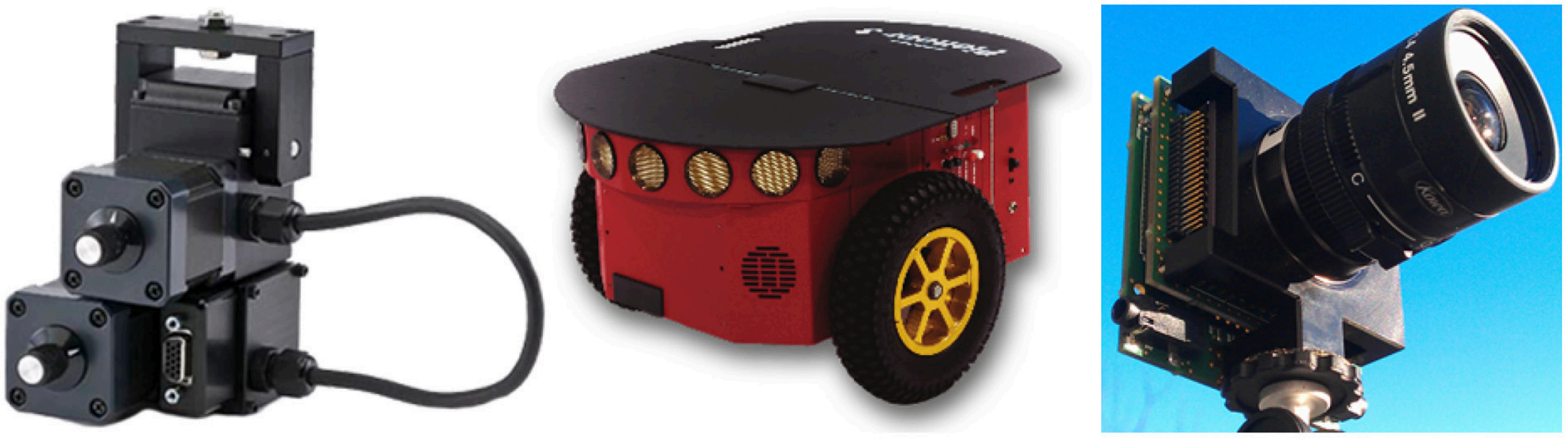
**Left:** Pan-Tilt Unit FLIR PTU-46-17P70T at http://www.flir.com/mcs/view/?id=53707. **Center:** Pioneer 3DX Mobile Robot at http://www.mobilerobots.com/ResearchRobots/PioneerP3DX.aspx. **Right:** DAVIS240b sensor at http://inilabs.com

The DAVIS sensor (Brandli et al., 2014) and a Microsoft Kinect Sensor (providing an RGB image and depth map) are mounted on a stereo rig and the stereo rig is mounted on a Pan Tilt Unit (PTU-46-17P70T by FLIR Motion Control Systems) on-board a Pioneer 3DX Mobile Robot. The motion is due to the rotation of the PTU defined by pan and tilt angles and angular velocities, and the translation of the Pioneer 3DX Mobile Robot defined by the direction of translation and the speed. ROS (Robot Operating System) packages are available for both the PTU and the Pioneer 3DX mobile robot. Figure 1 shows the Pan Tilt Unit on the left, the Pioneer 3DX mobile robot in the center, and the DAVIS sensor (a DAVIS240b by Inilabs) on the right.

Our dataset provides the following:

- *The 3D motion parameters: 3D translation and 3D pose of the camera*. These are provided by the PTU and the Pioneer Mobile Robot. Calibration of the PTU with respect to the platform, and calibration of the DAVIS with respect to the PTU are required.
- *The image depth* in the coordinate system of the DAVIS. Depth is obtained by the Microsoft Kinect Sensor (RGB-D sensor). A stereo calibration registering the Kinect depth to the DAVIS camera coordinates is required.
- *The 2D motion flow field*. Using the 3D motion and depth, the 2D motion flow field in the DAVIS coordinate system is computed.

### 3.3. DAVIS and RGB-D sensor calibration

The RGB-D sensor provides the depth of the scene. This depth needs to be transformed to the coordinate system of the DAVIS. In our procedure, we first calibrate the two cameras individually, both for intrinsic and extrinsic parameters. Next, since the spatial resolutions of the two cameras are very different, we compute the transformation of the depth by creating an intermediate 3D model from the Kinect data, which subsequently is projected to the DAVIS coordinate system.

In the very first step the RGB data and the Depth of the Kinect, which internally are captured by two separate sensors, are aligned to each other using the Kinect SDK. Next, the Kinect intrinsic and extrinsic sensor camera parameters are obtained using conventional image camera calibration on RGB data. Similarly, the DAVIS intrinsic and extrinsic camera parameters are obtained using conventional image camera calibration on the APS frames of the DAVIS (the APS frames and the DVS events in the DAVIS are geometrically calibrated). However, we note that the DVS event signal of the DAVIS, may also be calibrated by itself using a calibration grid of flashing LEDs (Mueggler et al., 2015). Such a procedure can be used if only a DVS (but not a DAVIS) is available. We can use the procedure of Mueggler et al. (2015), which consists of two steps: first it adjusts the focus, then is computes the intrinsic parameters. The code is based on ROS, and the calibration uses OpenCV functions.

The second step involves first a stereo calibration between the RGB-D sensor and the DAVIS, which provides the rotation and translation of the two sensors with respect to each other. Then the depth between the two cameras is registered via a 3D world model. In detail, the procedure involves the following transformations.

First, the Kinect 2D image coordinates are compensated for radial distortion as:

(1) $$x^{\prime }=x(1+k_{1}r^{2}+k_{2}r^{4}+k_{3}r^{6})$$

where *k*_1_, *k*_2_, *k*_3_ are the radial distortion coefficients, **x** and **x'** are the distorted and undistorted image coordinates respectively, and *r* = ∥**x**∥.

Next the 3D world coordinates **X**_**w**_ = (**x**_**w**_, *z*_*w*_) are obtained from the 2D image coordinates, **x'**, as:

(2) $$\left[\begin{array}{c} X_{w}\\ z_{w} \end{array}\right]=\left[\begin{array}{c} -(x^{\prime }-c)z\frac{1}{f}\\ z \end{array}\right]$$

where **c** denotes the principal point, *f* the focal length of the Kinect camera, and *z* the depth.

The 3D point cloud is then transformed using the geometric transformation between the sensors, given by the 3 × 1 translation **t** and 3 × 3 rotation *R* obtained by the stereo calibration. The transformation is formulated as **X'**_**w**_ = *R***X**_**w**_ + **T**, where **X'**_**w**_ is the new point cloud model in the 3D world.

**Figure 2:**
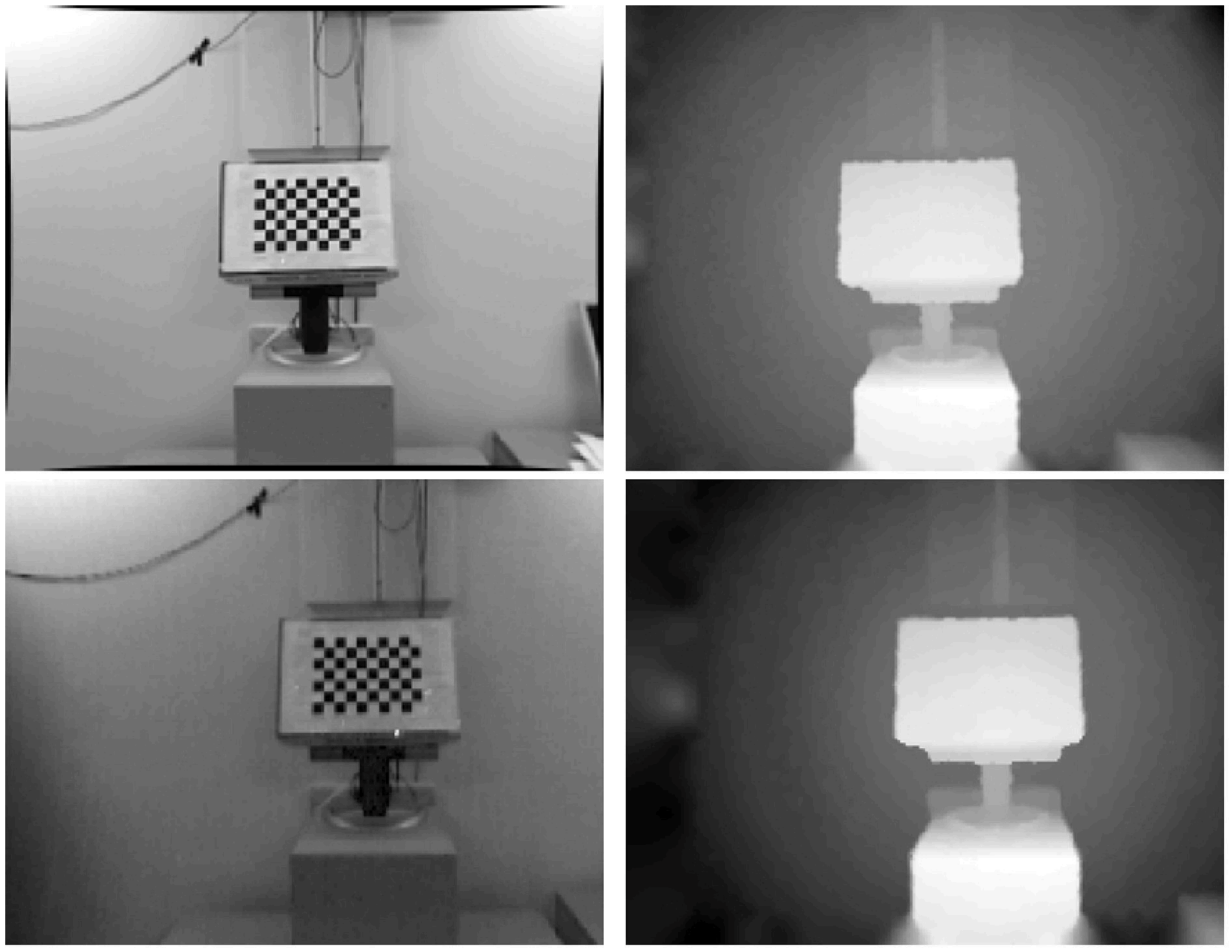
**Depth registration from RGB-D sensor (top row) to DAVIS sensor (bottom row)**.

Lastly, the point cloud **X'**_**w**_ is projected onto the 2D sensor plane of the DAVIS to obtain the sensor coordinates **x**_**D**_ as:

(3) $$x_{D}=x_{w}\frac{f_{D}}{z_{w}}+c_{D}$$

where **c**_**D**_ denotes the principal point and *f*_*D*_ the focal length of the DAVIS sensor. The depth for each image coordinate in the DAVIS image plane is registered using the Z-buffer. Any holes or ambiguities in the new registered depth are filled in using the inpainting method in Janoch et al. (2013), which assumes second order smoothness, minimizing the curvature in a least-squares manner. An example of the result of this calibration is shown in Figure 2.

### 3.4. DAVIS sensor and PTU calibration

**Figure 3:**
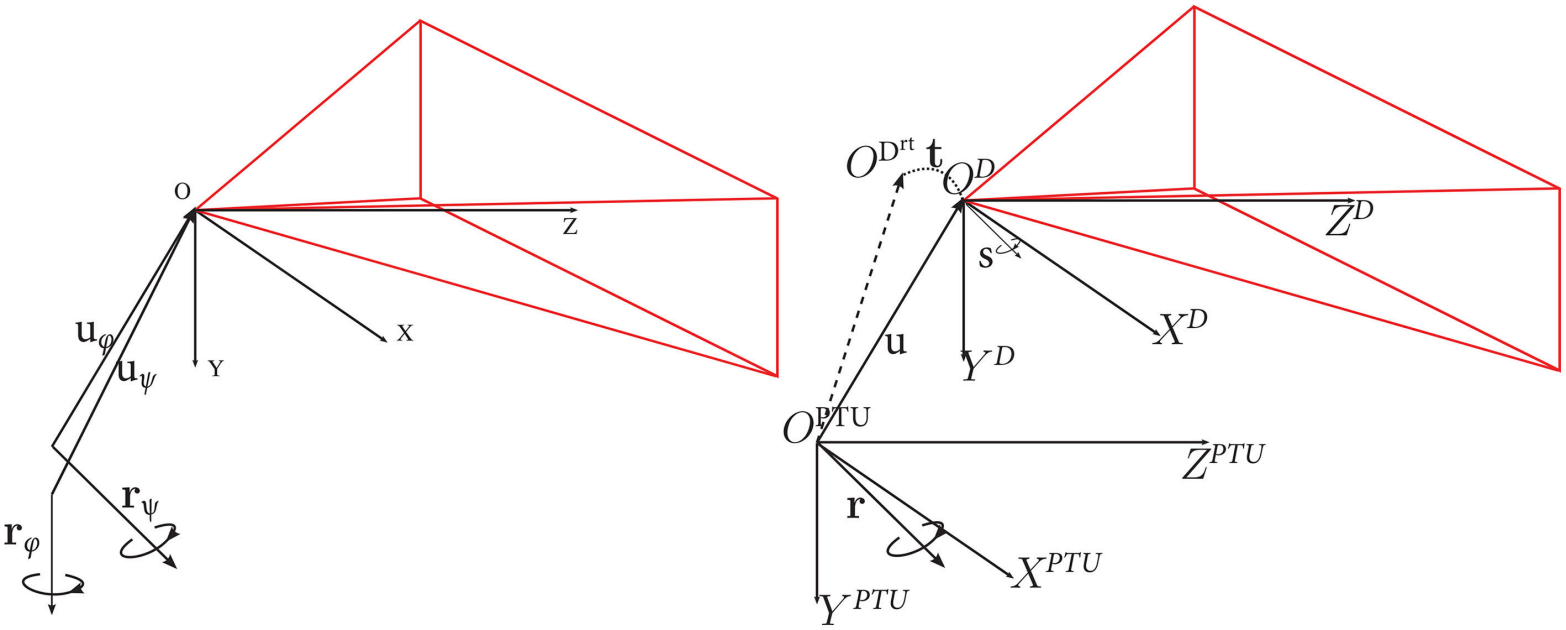
**Left:** Translation vector **u** of the DAVIS coordinate system with respect to the PTU, and **r**, the PTU rotation axis. The pose of the DAVIS sensor is represented by its axis **s**. **Right:** DAVIS coordinate system *O*^*D*^ and PTU coordinate system *O*^*PTU*^. *O^D^rt^^* represents the DAVIS coordinate system after a pan-tilt rotation of the PTU, characterized by a translation **t** and the rotation *R* around its axis **r**. Image adapted from Bitsakos (2010).

This section explains how to obtain an analytic expression for the rotation *R*_α_ and translation *T*_α_ of the DAVIS sensor (in its coordinate system) corresponding to a pan or tilt angle α of the PTU. This is a non-trivial task. The procedure is as follows: We first derive the translation and rotation for a number of pan-tilt combinations with respect to a base pose (pan = 0°, tilt = 0°) in the DAVIS camera. Then, we use these derived values to compute the (fixed) transformation between the DAVIS coordinate system and the PTU coordinate system. The parameters involved are the translation **u** between the coordinate systems, the rotation axis **r** of the pan-tilt unit, and the rotation axis **s** of the camera (see Figure 3).

First, we derive the translation and rotation of the DAVIS corresponding to various pan (rotation in the horizontal plane) and tilt (rotation in the vertical plane) combinations. In order to do that, we capture APS images with the DAVIS sensor for a number of pan tilt combinations, and perform a stereo calibration for each set of images with respect to the baseline (pan = 0° and tilt = 0°). We use as angle rotations for pan and tilt the values [−5°, −4°, −3°, −2°, −1°, 0°, 1°, 2°, 3°, 4°, 5°]. Since the transformation for pan and tilt can be applied independently, we do not need different combinations of pan and tilt. Thus, we have 11 pan combinations (0° tilt, including the base-pose, pan = 0° and tilt = 0°) and 10 tilt combinations (0° pan). For every combination, we take 10 images for the calibration, each with a different pose and position of the calibration pattern. The calibration provides the extrinsic rotation and translations of the DAVIS coordinate system with respect to the base-pose.

Let us now compute the *translation* of the DAVIS sensor center with respect to the PTU center. Consider the center of the coordinate system of the DAVIS for the baseline position *O*^*D*^. The position of the coordinate center for a combination of pan and tilt *O^D^rt^^* is described by a translation **t** with respect to the center of coordinates of the baseline *O*^*D*^. This translation **t** corresponds to the extrinsic translation estimated in the calibration of a pan-tilt-combination with respect to the baseline (explained in the previous paragraph). The camera center *O*^*D*^ is described by a translation **u** with respect to the PTU coordinate center, and a rotation *R* moves it to position *O^D^rt^^* (see Figure 3). Thus, we have in the coordinate system of the PTU that:

(4) $$\begin{array}{l} O^{D^{rt}}=R\;\cdot \;u\\ O^{D^{rt}}=u+t \end{array}$$

Note that there are multiple combinations of pan and tilt rotations (for different angles θ), and thus multiple *R* and **t**. The *R* for a specific angle θ can be re-written with respect to its axis **r** (in this case, only two variables), using the Rodrigues formula as:

(5) $$R=(1-cos(\theta ))K^{2}+sin(\theta )K+I$$

where *K*^2^ = **r** · **r**^*T*^ − *I*. Now, substituting *Rs* from Equation (5) into the equality resulting by combining the two constraints of Equation (4), and taking into account that the system has a total of *N* combination angles, the following minimization problem is formulated:

(6) $$\underset{r,u}{\arg\min}\sum_{i\in [1,\ldots ,N]}\|((1-cos(\theta_{i}))(r\cdot r^{t}-I)+sin(\theta_{i})K)\cdot u-t_{i}\|$$

where the rotation axis is a unit vector, i.e., ∥**r**∥ = 1.

**Figure 4:**
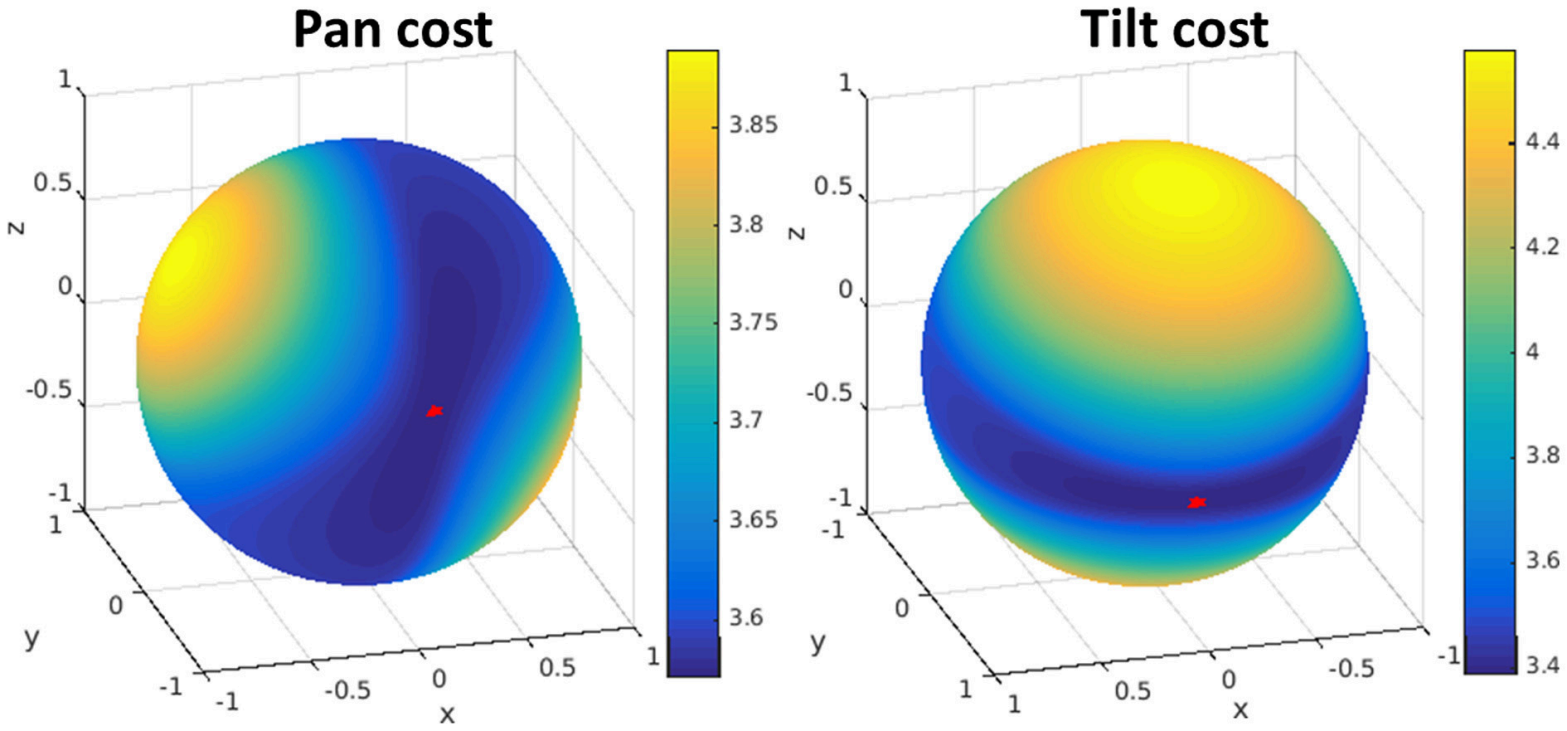
**Visualization of the error function from the minimization for pan (left) and tilt (right)**. The minimum error is marked on the sphere with a red star. The search is done in spherical coordinates over the rotation axis *r*, which has 2° of freedom. For each rotation we solve for the (best) translation.

The minimization with respect to the rotation axis **r** and the translation **u** is non-convex. However, the problem can be solved searching for the rotation axis and solving for the translation, using the interior-point method. Since the rotation axis has only 2° of freedom, we use a change of variables to search over spherical coordinates as in Bitsakos (2010). The minimization cost for our stereo rig is shown in Figure 4 where the minimum is marked on the sphere with a red star.

The second part computes the *rotation* axis **s** of the DAVIS sensor coordinate system. Since the rotation vectors derived for positive and negative angles of pan and tilt were found of nearly same value (but different sign), we did not formulate another minimization, but estimated the axis by taking the average of the values for the first two components. Using the fact that **s** is a unit vector provides the third value.

Finally, we obtain the following expression to compute for a given pan or tilt angle α the corresponding rotation *R*_α_ and translation *T*_α_ in the DAVIS sensor coordinates:

(7) $$T_{\alpha }=((1-cos(\alpha ))(r\;\cdot \;r^{t}-I)+sin(\alpha )K)\;\cdot \;u$$

(8) $$R_{\alpha }=(1-cos(\alpha ))L^{2}+sin(\alpha )L+I$$

where *L*^2^ = **s** · **s**^*t*^−*I*. Please note that the rotation and translation of the DAVIS coordinate system is applied independently to pan and tilt rotations, and we have two different rotations and translations for pan and tilt angles, respectively (denoted as θ and ϕ in Figure 3).

Finally, the motion of the Pioneer 3DX Mobile Platform is always a translation in the horizontal plane in the direction of Z. For our case, we considered the coordinate centers of the Pioneer and the PTU to be aligned. Thus, the translation of the mobile platform can be directly applied to the DAVIS sensor.

The code for the extrinsic and intrinsic calibration of the DAVIS and the RGB-D sensors, their stereo calibration, and the calibration between the DAVIS and the Pan-Tilt Unit is provided along with the dataset.

### 3.5. Generation of motion flow fields

The image motion flow field is the projection of the velocities of 3D scene points onto the image plane. Assuming a rigid motion [with translational velocity **t** = (*t*_1_, *t*_2_, *t*_3_) and rotational velocity **w** = (**w**_1_, **w**_2_, *w*_3_)] , the 3D instantaneous motion **Ṗ** of scene points **P** = (*X, Y, Z*) is given as **Ṗ** = −**t** − **w** × **P** (Longuet-Higgins and Prazdny, 1980). Then the equations relating the velocity (*u, v*) at 2D image points (*x, y*) to the 3D translation and rotation and the depth *Z* amounts to:

(9) $$u(x,y)=\frac{1}{Z}(-t_{1}f+xt_{3})+w_{1}\frac{xy}{f}-w_{2}\left(\frac{x^{2}}{f}+f\right)+w_{3}y$$

(10) $$v(x,y)=\frac{1}{Z}(-t_{2}f+yt_{3})+w_{1}\left(\frac{y^{2}}{f}+f\right)-w_{2}\frac{xy}{f}-w_{3}x$$

## 4. Evaluation methodology

The metrics we use to evaluate event-driven methods are similar to the ones previously used for frame-based techniques. Image motion flow fields will be evaluated using the average endpoint error (Otte and Nagel, 1994; Baker et al., 2011), which is defined as the average value of the vector distance between the estimated motion **u** and the ground-truth $\hat{\text{u}}$, and is derived for *N* motion flow values as:

(11) $$AEPE=\frac{1}{N}\sum_{i=1}^{N}\left\|u_{i}-{\hat{u}}_{i}\right\|.$$

Another representative metric, the average angular error (AAE), measures the average angular distance as:

(12) $$AAE=\frac{1}{N}\sum_{i=1}^{N}\arccos\left(\frac{{\hat{u}}_{i}^{t}u_{i}}{\left\|{\hat{u}}_{i}\|\|u_{i}\right\|}\right).$$

We provide the code for computing the AEPE and AAE of a motion flow field. Similarly, we evaluate 3D camera motion (given by 3D rotation and translation vectors) as averages using the same measures, but in this case averaging over time.

In order to evaluate the robustness of motion flow field estimation, we provide the R*X* value (Scharstein and Szeliski, 2002), which measures the percentage of estimates with an error above *X*. So the larger the value, the worse the motion estimation. In the Middlebury (Baker et al., 2011) evaluation, this measure is used with the endpoint error for R *0.5*, R *1.0*, and R *2.0*. To evaluate the significance of the computed measure, we also provide a statistical significance test. We use the Wilcoxon signed rank test (Wilcoxon, 1992), for which a *p* < 0.05 shows statistical significance (see also Roth and Black, 2005; Sun et al., 2014).

Different from frame-based flow, the flow from event-driven techniques is sparse. We also provide a measure for the sparseness of the estimation. The so-called density value expresses the percentage of motion estimates within a fixed time interval. In Computer Vision, although not common, optical flow density is considered in some works (see e.g., Barron et al., 1994; Brandt, 1997; Barranco et al., 2012).

## 5. Dataset examples for DAVIS sensor mounted on the robotic platform

We recorded more than 40 sequences of diverse scenarios, with the camera mounted on a Pan-Tilt unit on-board the Pioneer 3DX Mobile Platform. All the sequences are due to rigid 3D motions: pure pan or tilt motion, combined pan and tilt motion, translation of the robotic platform only (forward or backward translation), and combinations of pan, tilt, and translation. The scenes are from an an office and have a variety of objects of different sizes and shapes, such as chairs, tables, books, and trash bins. Texture was added to some of the objects to obtain a higher DVS event density. The depth is in the range of ~0.8–4.5 m (also constrained by the use of Kinect), and the motion flow between frames (at about 50 ms apart) is up to 5–10 pixels. There are a variety of rigid motions, including sequences that are mostly due to rotation, sequences that are mostly due to translation, and sequences with balanced rotation and translation.

**Figure 5:**
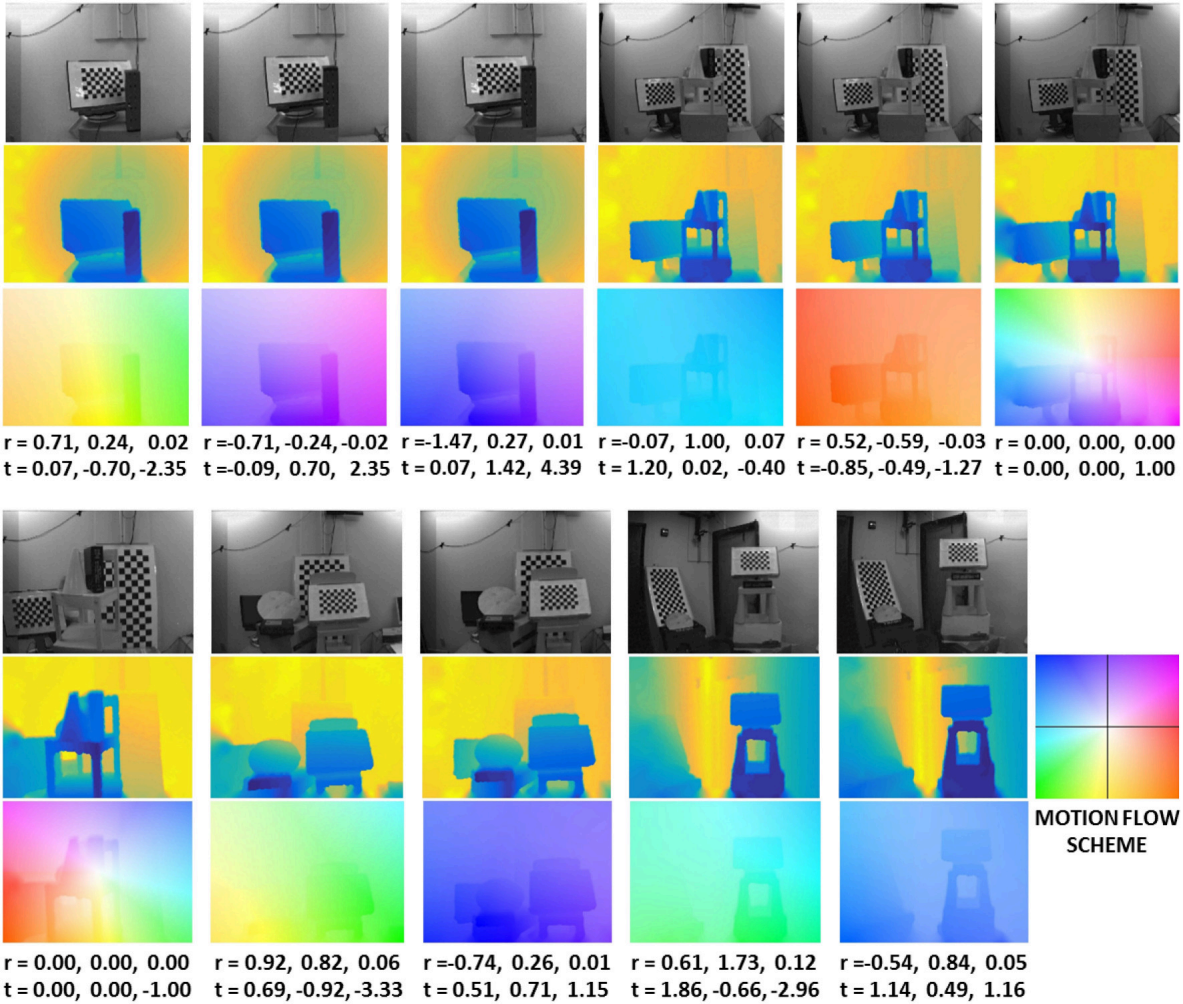
**Example sequences from the dataset**. For each sequence we show: DAVIS APS frame (first row), depth map (second row), motion flow field (third row), and the rotation and translation values (in 10^−2^ rad/frame and 10^−2^ pix/frame). The color coding for the depth map uses cold colors for near and warm colors for far points. The motion flow fields are color-coded as in Baker et al. (2011), with the hue representing the direction of motion vectors and the saturation their value.

Figure 5 shows a few of the sequences. The first row shows the DAVIS images, the second the depth maps, and the third the motion flow fields (using the color-coding of Baker et al., 2011). The first group of five images is from a pan and tilt motion, the last image on the top right and the first at the bottom left are from a pure zoom in and zoom out motion, respectively. The last group at the bottom are from combined pan tilt and zoom in or zoom out motions, and the scenes are cluttered with objects of different shapes and at different depth ranges. The six parameters for the rotation and translation are shown below the figures. The complete dataset is available at the website.

## 6. Conclusions

We presented the first datasets for evaluating techniques of visual navigation with neuromorphic sensors. These datasets contain synthetic and real sequences of rigidly moving sensors in static environments. The data, which we provide, includes the images, the event streams, the 3D depth maps, and the 3D rigid motion of the sensor. Using these datasets one can evaluate and compare event-based and classic frame-based techniques of image motion estimation, 3D motion estimation, scene reconstruction, and segmentation by depth. We also provide the code for the various calibration procedures used in order to facilitate future data collection and code for evaluation.

We plan to maintain the website, and add new more challenging sequences including a larger variation of scenes and dynamic scenes in the future. We also plan to evaluate and publish the results of different methods. So far we used the same evaluation metrics as in Computer Vision, which only address the accuracy of estimation. Since currently there are very few techniques available, the efficiency of computation on events has not been addressed yet. However, as new neuromorphic methods will be developed, and it becomes useful to evaluate and compare algorithms, we will also need to develop evaluation criteria aimed at the complexity of computation.

## Author contributions

FB performed the experiments and data analysis, and drafted the manuscript. CF, YA, and TD drafted the manuscript and performed data analysis.

## Funding

This work was supported by an EU Marie Curie grant (FP7-PEOPLE-2012-IOF-332081), the EU Project Poeticon++ under the Cognitive Systems program, the National Science Foundation under grant SMA 1248056, grant SMA 1540917, and grant CNS 1544797, the Junta de Andalucia VITVIR project (P11-TIC-8120), and by DARPA through U.S. Army grant W911NF-14-1-0384.

### Conflict of interest statement

The authors declare that the research was conducted in the absence of any commercial or financial relationships that could be construed as a potential conflict of interest.
